# A Perceptual Motor Intervention Improves Play Behavior in Children with Moderate to Severe Cerebral Palsy

**DOI:** 10.3389/fpsyg.2016.00643

**Published:** 2016-05-03

**Authors:** Brigette O. Ryalls, Regina Harbourne, Lisa Kelly-Vance, Jordan Wickstrom, Nick Stergiou, Anastasia Kyvelidou

**Affiliations:** ^1^University of Nebraska at Omaha, OmahaNE, USA; ^2^Duquesne University, PittsburghPA, USA; ^3^College of Public Health, University of Nebraska Medical Center, OmahaNE, USA

**Keywords:** cerebral palsy, biomechanics, intervention, play, physical therapy, children, motor development

## Abstract

For children with moderate or severe cerebral palsy (CP), a foundational early goal is independent sitting. Sitting offers additional opportunities for object exploration, play and social engagement. The achievement of sitting coincides with important milestones in other developmental areas, such as social engagement with others, understanding of spatial relationships, and the use of both hands to explore objects. These milestones are essential skills necessary for play behavior. However, little is known about how sitting and play behavior might be affected by a physical therapy intervention in children with moderate or severe CP. Therefore, our overall purpose in this study was to determine if sitting skill could be advanced in children with moderate to severe CP using a perceptual motor intervention, and if play skills would change significantly as sitting advanced. Thirty children between the ages of 18 months and 6 years who were able to hold prop sitting for at least 10 s were recruited for this study. Outcome measures were the sitting subsection of the Gross Motor Function Measure (GMFM), and the Play Assessment of Children with Motor Impairment play assessment scale, which is a modified version of the Play in Early Childhood Evaluation System. Significant improvements in GMFM sitting scores (*p* < 0.001) and marginally significant improvement in play assessment scores (*p* = 0.067) were found from pre- to post-intervention. Sitting change explained a significant portion of the variance in play change for children over the age of 3 years, who were more severely affected by CP. The results of this study indicate that advances in sitting skill may be a factor in supporting improvements in functional play, along with age and severity of physical impairment.

## Introduction

“The work of children is play." This often repeated saying encapsulates the idea that the active engagement of a child in exploring, investigating, experimenting, and experiencing the world, also known as “playing," contributes to the development of physical, emotional, social, and cognitive development. Engagement in play, particularly complex exploratory and pretend play, is a central activity of early childhood and is linked to the development of cognition, language, problem solving, and social skills (Piaget, 1951; Fewell and Rich, 1987; Singer and Singer, 1990; Hughes, 1991; Farmer-Dougan and Kaszuba, 1999; Russ, 2003; Singer et al., 2006; Bagnato, 2007; Orr and Geva, 2015). Sitting, on the other hand, is an essential motor skill that allows the infant to view and interact with the world in a completely different way and promotes more complex play activities. In the present study, we were interested in the relationship between the development of play and the development of sitting in children with motor impairments. Specifically, we explored whether improvements in a child’s ability to sit influences his/her ability to engage in play.

Spontaneous, self-directed play in early childhood, as traditionally characterized (Piaget, 1951; Vygotsky, 1980), requires the use of the hands to reach and interact with objects and toys. The emergence of sitting in typically developing infants at approximately 6 months of age coincides with many skills necessary for play, including improved accuracy in reaching (Rochat, 1992; Harbourne et al., 2013), increased understanding of the spatial properties of objects (Soska et al., 2010), and greater efficiency in visual attention to the environment (Harbourne et al., 2014; Surkar et al., 2015), among others. Sitting stability frees the arms for exploration and object manipulation, and allows the head and trunk to freely move and orient to important information in the environment (Rochat and Goubet, 1995). Sitting posture during reaching appears to rely more on anticipatory processes (Hadders-Algra, 2013). In addition, muscle activation patterns at the onset of sitting are highly variable, and as sitting and reaching develop, these patterns become gradually refined for both tasks (Harbourne et al., 1993, 2013; Hadders-Algra et al., 1996). Studies investigating the development of sitting postural control while reaching suggest that reaching may serve as a perturbation for the maintenance of postural control in infancy (Hadders-Algra, 2013; Harbourne et al., 2013), although hand use clearly increases as sitting develops (Rochat, 1992; Rochat and Goubet, 1995; Harbourne et al., 2013). Thus, evidence from research with typically developing infants indicates that emerging postural control serves to support the development of environmental exploration such that an infant’s ability to play and engage in the world improves, which may, in turn, lead to further cognitive advancement.

Although improving postural control may be related to increasing upper extremity skill, a causal relationship is not necessarily evident (Harbourne et al., 2013). Evidence to date reveals contradictory findings regarding the effect of postural control on reaching or play behavior in typically developing infants and infants with developmental delays. Investigations of the specific relationship of proximal (or postural) control to distal (or hand) control do not support the tenet that improving postural control must precede advances in hand skill in the developing child (Loria, 1980; Fetters, 1991). A recent analysis of gross motor function to upper extremity control in children with CP concluded that there was a poor overall correlation between the two, and that the relationship varied between subtypes of CP (Carnahan et al., 2007). In infants with neuromotor impairments, the short-term effect of using a supportive seat to control posture led to no immediate improvement in object manipulation (Washington et al., 2002). On the other hand, providing support at the pelvis in typically developing infants that cannot achieve sitting independently enhanced the coordination between trunk control and reaching (Rochat and Goubet, 1995). Reports from parents have indicated that specific adaptive seating enabled their children to participate more in play activities and address their self-care needs (Rigby et al., 2009) whereas the absence of these devices led to negative outcomes (Ryan et al., 2009). However, a recent systematic review suggested that there are more studies needed to investigate the linkage between sitting postural control and every day life activities (Angsupaisal et al., 2015). Thus, the relationship between sitting postural control and object exploration with the upper extremities cannot be considered as causal, although researchers have identified the co-emergence of the two skills.

Even if postural control influences reaching behavior in typically developing infants, little is known about the specific relationship between the development of sitting and play in children with motor disorders, particularly those with a moderate to severe condition. Poor postural control is associated with limitations in the attainment of functional skills such as mobility and manipulation during the developmental process. However, therapeutic intervention also targets postural control in order to affect upper extremity skill. Research has linked qualitative improvement in reaching with responsiveness to intervention of overall motor skill in children with severe CP (Fetters and Kluzik, 1996) as well as in typically developing infants (Rochat and Goubet, 1995; Out et al., 1998), but the nature of the connection between upper extremity function and postural control is still poorly understood. Adolph and Berger (2006) refer to the ‘centrality of posture’ as a necessary condition for looking and interacting with the environment around them. However, there are no studies that investigate how the development of sitting postural control would affect play behavior and interaction with objects in children with cerebral palsy (CP) who have not developed sitting independence. Thus, it is important to understand how improvements of sitting postural control ability might influence play behavior in children with CP because play skills reflect the problem-solving skills necessary for independent function.

The prevailing method in physical therapy intervention of children with CP is Neuro-Developmental Treatment (Bobath, 1971). This method emphasizes the reduction of abnormal muscle tone and the facilitation of normal postural reflexes. Assisted movement in specific patterns is encouraged to normalize muscle tone. Facilitation of more normal movement is a primary focus, and it is done through graded stimulation at certain key points of the body (Trahan and Malouin, 2002). Normal postural alignment is emphasized in this approach. A recent review of the body of evidence regarding this intervention approach found little support for its effectiveness in promoting normal motor milestones in any type of condition (Butler and Darrah, 2001; Novak et al., 2013). For this reason we chose a different intervention for the present project.

An alternative approach that is based on perception-action theory is the perceptual motor intervention of Tscharnuter (1993, 2002). This method emphasizes the ecological approach and spontaneous movement based on environmental affordances. Self-initiated, functionally directed movement drives the focus of intervention. This intervention consists of activities that include handling, which gently calls the child’s attention to the support surface, and sets up the environment for small increments of movement that the child can utilize to solve a movement problem. Passive movements are not used in this approach. Increased variability of active movement is encouraged, and movements that may be considered abnormal in other approaches are not blocked or discouraged. This perceptual motor approach was used as one of the interventions for a previous project, with preliminary evidence of effectiveness to improve postural control over and above a home program (Stergiou et al., 2006; Harbourne et al., 2010).

Because infants and children with severe motor impairments such as CP are often limited in their ability to manipulate objects (Duff and Charles, 2004; Arnould et al., 2008), measuring and assessing play is a challenging task. Prior to this study, no play-based assessment system had been adapted for use with severely motor impaired children. In the present study, we used a new scale, the Play Assessment of Children with Motor Impairment (PACMI) Scale^1^ The PACMI is a modified version of the Play in Early Childhood Evaluation System (PIECES) developed by Kelly-Vance and Ryalls. The PIECES has been empirically documented to be both a valid and reliable measure of play in typically and atypically developing children (Kelly-Vance et al., 1999, 2002; Kelly-Vance and Ryalls, 2005). As described in Section “Materials and Methods,” the coding scheme used in the PIECES was expanded in order to capture basic play manipulation behaviors at a fine-grained level. These play behaviors included both successful and unsuccessful child-initiated attempts to manipulate toys.

In summary, the primary goal of the present study was to help fill a gap in the literature by directly examining the relationship between improvements in sitting and a child’s ability to engage in spontaneous play after a perceptual motor intervention in children with moderate and severe CP. We had two specific questions. First, we examined if sitting skill could be advanced in children with significant motor impairments using an intensive perceptual motor intervention. Second, we questioned if children’s play skills would change as sitting ability advanced and whether improvements in sitting would be associated with improvements in the complexity of play. Our hypothesis was that the intervention would improve sitting and play ability and that the changes in sitting ability would explain a significant proportion of the variance in the change of play scores.

## Materials and Methods

### Participants

Participants were 30 children with moderate (*N* = 12) to severe (*N* = 18) CP. All children were between the ages of 18-months and 6-years (11 female, 19 male). Children were recruited from a group of children who participated in a previous study, from the University of Nebraska Medical Center community, and by word of mouth. Procedures were approved by the Institutional Review Board of the University of Nebraska Medical Center, and consent was obtained from the parent(s) of each child before participating.

To be included in this study, children were required to have a diagnosis of CP and be unable to sit independently. In order to assess the distribution of children with moderate and severe CP, we used a scale created in a previous study of infants with CP (Harbourne et al., 2010). Beginning sitting skills were required for entry into the study. We defined beginning sitting as: the ability to prop sit while floor sitting for at least 10 s when placed; the ability to hold the head in line with the body (not falling forward) while prop sitting; when supported by another person in the sitting position, the child is able to move the arm toward a person or toy, but does not need to grasp the toy. Children were excluded from participation in this study if they had a diagnosis of blindness, a diagnosed hip dislocation or subluxation of the hip over 50%, or an additional diagnosis that affected his/her neuromuscular system (e.g., Down syndrome or spina bifida).

### Measures

#### Play Assessment of Children with Motor Impairment

Assessing the play skills of young children with severe motor impairments, such as CP, is challenging because of their limited ability to manipulate objects (Duff and Charles, 2004; Arnould et al., 2008). Several measures exist but none are tailored to the unique needs of these children. Therefore, an expanded version of the PIECES was used to assess the participants’ play skills (Kelly-Vance and Ryalls, 2014). The PIECES was developed based on thorough research and theory on play across developmental stages and has been shown to have high psychometric properties with an interrater reliability of 90% for typically developing children and as high as 100% for children with exceptionalities and moderate test–retest correlations for each population (*r* = 0.48 and *r* = 0.58, respectively; Kelly-Vance and Ryalls, 2005). The PIECES is an observation of a child’s free play with toys that results in a description of exploratory and pretend play skills. The scale has been used with children who have a variety of exceptionalities including motor impairments, autism spectrum disorder, and speech/language impairments (Kelly-Vance and Ryalls, 2014). This scale was selected as the play measure because it could be adapted to the needs of the children in this study.

The expanded version of the scale is called the PACMI. It was derived from the exploratory play scale of the PIECES that included an assessment of a child’s ability to explore toys by mouthing, manipulating, and discovering their function^1^. Due to the limited motor ability of the participants in the present study, most of the children were unable to play with toys in the same manner as typically developing children. Typically developing children use their hands to explore toys, but due to the limited motor skills of children with CP, a more general definition of toy manipulation was used. Children could initiate exploratory play by successful manipulation (SM), proximal manipulation (PM), or unsuccessful manipulation (UM). SM includes using any body part to manipulate a toy, such as pressing a play piano key with one’s finger or forehead and resulting in an audible note. PM involves using a body part in close proximity to the toy without any attempt to manipulate the toy. This would occur if the child puts a hand on the piano but does not press an individual key. UM is when the child makes an attempt to manipulate a toy but is not successful. An example of UM is when a child puts a finger on a piano key but is unable to press it down.

The overall result of the play assessment conducted in this study was a Self-Initiated Play Composite (SIPC) score, which was computed by adding all SMs, PMs, and UMs and then dividing the total number by the overall time spent with the toy. High inter-observer reliability was found on the PACMI (see Procedure section) which is consistent with findings on the overall PIECES.

#### Gross Motor Function Measure-88

The Gross Motor Function Measure-88 (GMFM) was used to evaluate changes in sitting skill over time. This measure was designed for use with children with CP, and evaluates motor skills in five areas: lying and rolling, sitting, crawling, standing, and walking/running/jumping. It took approximately 20 min to administer the test, with time varying according to the ability level of the child and his/her cooperation and understanding. This scale has been validated in children 5 months to 16 years-old (Russell et al., 1993). We utilized only the sitting subsection for this study.

### Procedure

Each child received 45 min of physical therapy intervention twice a week for 12 weeks. The intervention received by the children was performed by therapists trained in perceptual motor techniques that are based on the approach of Tscharnuter (1993, 2002). In general, the approach utilizes environmental forces during self-initiated goal-directed movements to change function and postural control. The specific techniques used during intervention were dependent on the skill level and interest of the child. Overall, activities were aimed at teaching the child to attend to significant environmental information, such as pressure against the support surface, which can be correlated to forces useful for controlling posture and movement, and all activities were related to interaction with objects of interest to the child. We allowed the child to choose the movement strategy even if the movement appeared atypical, thus allowing for child-initiated movement. The therapist presented an environmental modification requiring a small movement or postural challenge to the child, and waited for the child to solve the problem, giving very light cues or assistance. The focus was on helping the child utilize forces to obtain a functional goal through problem solving. Fidelity of the approach was maintained by having only three therapists who were trained in the approach provide the intervention, under the supervision of a primary therapist. (For more information on the perceptual motor intervention refer to Harbourne et al., 2010.)

Sitting (GMFM) and play (PACMI) data were collected at the Infant lab at the Munroe-Meyer Institute of the University of Nebraska Medical Center. This lab is designed to look like a home living room, with carpeted floor and living room furniture (e.g., a couch and end tables). Data was collected at two different times during the child’s participation in the study. The first session was at pre-test, prior to the child receiving any intervention sessions. The post-test session was conducted after the completion of the intervention, approximately 12 weeks later. For all data collection sessions, the children were allowed time to adjust to the setting.

Both play and sitting data were collected on the same day. The play assessment was conducted before the sitting assessment. Cameras were set up to record both sessions and all sessions were coded from the videotapes. To ensure consistency across sessions, a specific toy set was utilized. The toy set included a baby doll, a piano, a pop-up toy, a pull-toy, a telephone, pretend food items, a jack-in-the-box, and a toy car with people. A set of four to eight toys was used in each session, and four toys were set out for the child at a time. The goal was for the child to use all eight toys, but some children did not have the hand skill or interest level to play with all of the toys. The parents/caregivers were asked if the child would be interested in playing with a specific toy, and if the response was negative, that toy was eliminated from the set. A minimum of four and a maximum of eight toys were presented, per child, and the same toys were used at the post-test as at the pre-test. The examiner asked the child which toy she/he wanted to play with and the child was allowed to make the selection by scooting toward the toy, gazing at it, or reaching for it. If the child did not select a toy, the examiner did, one at a time, and presented it to the child. During the play assessment, most children were seated on the carpeted floor with a therapist seated behind them. The therapist provided the support needed, depending on the child’s sitting skill. Only as much support as needed was provided. One child, with severely limited sitting ability, was seated in her wheelchair for the pre- and post-test play assessment because the parent thought it was the best option for her. The play assessment took approximately 15 min.

To code the play assessment, two graduate assistants watched the session videotapes and provided a running description of what the child and the examiner did with the toys. These behavioral descriptions were then coded using the SIPC scale of the PACMI coding scheme. The percent of time that the child spent engaged with the toy was also calculated. Inter-rater reliability was calculated on 20% of the tapes, and an inter-observer correlation was found ranging from 0.97 to 0.99.

All statistical analyses were performed with SPSS software (version 16.0). The alpha level was set at 0.05. Paired *t*-tests were performed for the GMFM scores and the PACMI play scores between pre and post intervention. We also performed Pearson r correlations to identify linear relationships between the changes in the variables of interest, as well as severity, and age. Lastly, we performed a stepwise multiple linear regression analysis to investigate the percentage of variance of the change in SIPC play scores that could be explained by the change in the GMFM scores and by the play scores pre-intervention.

## Results

**FIGURE 1:**
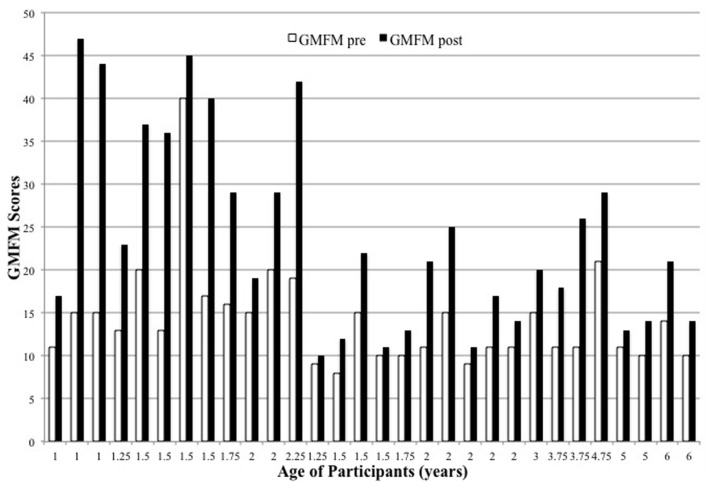
**Gross Motor Function Measure (GMFM) sitting scores between pre and post intervention**.

**FIGURE 2:**
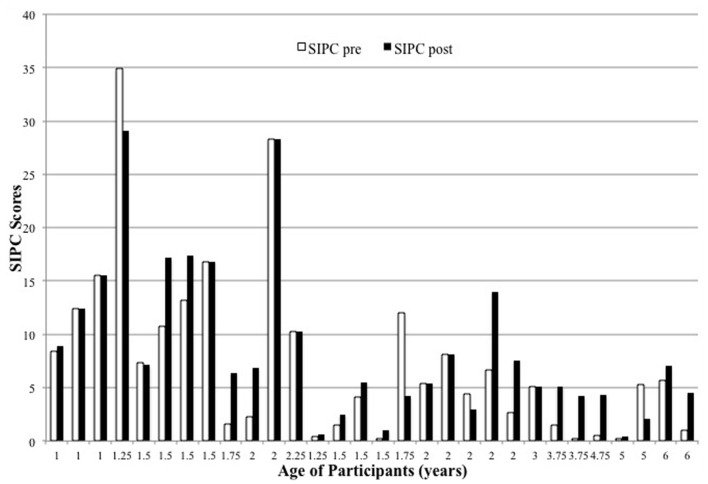
**Self-Initiated Play Composite (SIPC) scores between pre and post intervention**.

Descriptive data of the children’s age, GMFM and SIPC scores pre and post intervention are presented in **Table 1.** The children’s scores for sitting and play both pre- and post- test can be found in **Figures 1** and **2.** As can be seen in **Figure 1**, all children’s GMFM sitting scores improved from pre- to post-intervention. Statistical analysis indicated that the change was statistically significant [*t*(30) = -6.317, *p* < 0.001, effect size *r*: 0.761, Cohen’s *d*: -2.346]. As can be seen in **Figure 2**, a majority of children (18) showed improvements in their SIPC scores from pre- to post-intervention, although the effect was only marginally significant [*t*(30) = -1.903, *p* = 0.067, effect size *r*: 0.333, Cohen’s *d*: -0.706]. Moreover, it is important to note that seven children, although very delayed in motor skills, played in a cognitively advanced way. Different play strategies were noted that would be considered higher level, such as pretending the baby doll was real with hugs and kisses, rather than poking at the baby’s face. These advanced play strategies usually resulted in less repetitious behavior (counts of manipulation) but more social interaction with the parent, examiner, and toy item. By group consensus after viewing the play videotapes, we agreed that the scoring for the lower functioning children did not accurately represent the play behavior of the children who were more cognitively advanced. In those seven cases, we carried forward the pre-play score and made it their post-play score.

**Table 1:** Measures of central tendency of the main variables.

Variable	Minimum	Maximum	Mean	*SD*
Age	1	6	2.43	1.49
GMFMpre	8	40	14.20	6.03
GMFMpost	10	47	23.96	11.38
SIPCpre	0.20	34.90	7.56	8.15
SIPCpost	0.40	29.10	8.69	7.31


Gender initially appeared to be a significant factor. However, 25% of girls were in the moderate CP range, which appears as if gender was significantly related to our outcome measures. The primary composition of the moderate group was male. Although, we know that a disproportionate number of males are diagnosed with CP^2^, there is no data on gender differences by severity for the diagnosis of CP. We judged severity of CP to be more influential than gender by the composition of our groups, and verified our assumption with correlation and regression analysis. Finally, we used only severity and age in the regression models.

Bivariate correlations (**Table 2**) revealed that severity level was positively correlated with age (*p* = 0.004, older children were more severely affected by CP), and negatively correlated with GMFM pre (*p* = 0.005), post (*p* < 0.001) and change (*p* < 0.001) scores and SIPC pre (*p* < 0.001) and post (*p* < 0.001) scores. Age was negatively correlated with the SIPC pre (*p* = 0.036) and post (*p* = 0.041) scores. In addition, GMFM pre scores were positively correlated with the GMFM post (*p* < 0.001) and SIPC post (*p* = 0.018). GMFM post scores were positively correlated with the GMFM change (*p* < 0.001), SIPC pre (*p* = 0.015) and SIPC post (*p* = 0.002) scores. This result suggests that younger children had greater SIPC pre and post scores. The GMFM change score was positively correlated with the SIPC pre (*p* = 0.042) and SIPC post (*p* = 0.016) scores while the SIPC pre scores were positively correlated with SIPC post (*p* < 0.001) and negatively correlated with the SIPC change (*p* = 0.014) scores.

**Table 2:** Bivariate correlations.

Correlations									

		**Severity**	**Age**	**GMFMpre**	**GMFMpost**	**GMFMdiff**	**SIPCpre**	**SIPCpost**	**SIPCdiff**
Severity	*r*	1	0.506^∗^	-0.500ˆ*	-0.732ˆ*	-0.628ˆ*	-0.603ˆ*	-0.681ˆ*	-0.019
	*p*		0.004	0.005	0.000	0.000	0.000	0.000	0.920
Age	*r*		1	-0.134	-0.299	-0.307	-0.384ˆ*	-0.375ˆ*	0.119
	*p*			0.480	0.108	0.099	0.036	0.041	0.531
GMFMpre	*r*			1	0.687ˆ*	0.212	0.308	0.428ˆ*	0.190
	*p*				0.000	0.262	0.098	0.018	0.315
GMFMpost	*r*				1	0.856ˆ*	0.441ˆ*	0.550ˆ*	0.130
	*p*					0.000	0.015	0.002	0.494
GMFMdiff	*r*					1	0.374ˆ*	0.434ˆ*	0.039
	*p*						0.042	0.016	0.836
SIPCpre	*r*						1	0.917ˆ*	-0.444ˆ*
	*p*							0.000	0.014
SIPCpost	*r*							1	-0.049
	*p*								0.799
SIPCdiff	*r*								1


*^∗^signifies when there is a statistically significant correlation.*

Because age was positively correlated with severity, and after careful visual observation of the data we identified that 45% of children under age three were classified as severe, whereas 100% of the children over or equal to 3 years of age were classified as severe. Thus, we conducted an *ad hoc* stepwise linear regression analysis using age as the selection variable. We selected 3 years of age as our cut-off based on visual observation of the data. In addition, 3 years of age is the usual division in age for special services mandated by the Individuals with Disabilities Education Act; Part C differentiates services for children under the age of 3 (infants), from children over the age of 3 (school age; Adams et al., 2013). Thus, we ran two stepwise multiple linear regressions: one on children under three and one on children over three. First, we examined the proportion of variance explained by GMFM change, and SIPC pre scores on SIPC change scores in children less than age three (22 children). No significant relationship was found for these moderately delayed children. Second, we examined the proportion of variance explained by GMFM change, and SIPC pre scores on SIPC change scores in children greater or equal to age three (eight children). A significant proportion of the variance was explained [*F*(1,7) = 7.786, *p* = 0.027] with an *R*^2^ of 0.527 with only the GMFM change scores included in the model. Therefore, more than 50% of the variance in the SIPC change score was explained by the change in the GMFM scores for these older, more severely delayed children.

## Discussion

In the present study we had two primary goals. Our first goal was to document that an intervention grounded in perception-action theory (Tscharnuter, 1993, 2002) would improve sitting in children with moderate to severe CP. Our second goal was to examine whether children’s play skills would change as sitting ability advanced and whether the improvements in play were directly linked to improvements in sitting. Our results were positive with regard to the first goal and partially for the second goal, with the findings concerning the effect of the intervention on sitting being more straightforward than for the improvements in level of play. Specifically, analyses revealed that children with moderate or severe CP given a 12-week perceptual- motor intervention made significant gains in sitting ability and marginally significant gains in play behavior. With respect to our second goal our results revealed that improvements in play were directly linked to improvements in sitting only for children over 3-years of age. Thus, in this study, an improvement in sitting was linked to an improvement in play only for the severely impaired and older children. Specifically the results indicated that, for the older severely delayed children, a significant proportion of the variance in SIPC change scores was due to the change in GMFM scores from pre to post intervention.

There are several implications that can be drawn from the results of the present study. First, with respect to our first goal, we successfully documented the effectiveness of a perceptual motor intervention in sitting ability in children with moderate or severe CP. GMFM sitting scores of all the children that received the perceptual motor intervention improved, as shown in **Figure 1.** The perceptual motor intervention is based on the ecological approach and emphasizes spontaneous movement based on environmental affordances. Self-initiated, functionally directed movement is the focus of intervention. Perceptual motor intervention consists of activities that include handling, which gently drives the child’s attention to the support surface, and sets up the environment to produce small increments of movement that the child can utilize to solve a movement problem. Passive movements are not used in this approach. Increased variability of active movement is encouraged, and movements that are considered abnormal in other approaches are not blocked or discouraged. These results are in agreement with a younger cohort of children with CP who received the perceptual motor intervention in the first 2 years of life and improved sitting postural control (Harbourne et al., 2010). This is the first study to demonstrate that the specific perceptual motor intervention is effective in improving gross motor behavior in sitting in older children with moderate and severe CP. Fundamentally, perceptual motor experiences offer the opportunity for broad development and in other domains, such as social and cognitive development (Dusing et al., 2013; Lobo et al., 2013).

With respect to our second goal, the very design of the experiment presumed a link between motor behaviors such as sitting and a child’s ability to engage in play. The results of the experiment can be interpreted in this manner: the sitting intervention did not directly target play, and yet, overall, children both improved at sitting and most of them showed greater ability to manipulate the toys after the intervention. In typically developing children, the attainment of motor skills like sitting and reaching are temporally linked to the development of complex play behavior (Rochat and Goubet, 1995). However, further analyses revealed that improvements in play were only directly related to improvements in sitting for the older children in the study (3-years-old and above). The eight children that were above or equal to age three were in the severe CP range. With the exception of one child, all children maintained or improved their SIPC score as GMFM scores improved. However, for children less than 3-years-old, improvements in play were not correlated with improvements in sitting.

There are two possible reasons why we only found a significant linkage between sitting improvement and play change in the older children and not in the younger children. First, all the children in the study showed very delayed motor skills; all were at least 18 months of age and not yet sitting independently. Clearly, the older children were more severe simply when considering the discrepancy between their age and skill level. Thus, the more severe children had lower initial scores, and may have had more room to improve on the play scale. Second, the younger children advanced to a greater degree in motor skills, on average, during the intervention. Some of the younger children developed mobility, including the ability to get in and out of the sitting position. This new-found freedom to move appeared to take their interest rather than toy exploration, a phenomenon noted in typically developing children (Karasik et al., 2011). Infants who become mobile tend to have interest in distant objects, rather than objects close at hand, as in our play paradigm. Alternatively, the younger children with moderate CP may have had better inherent trunk and arm control, which did not show as large a degree of change during the intervention. Because we used only the sitting section of the GMFM, and not the crawling section, these children may have reached the zenith on the motor score they could achieve. This would influence the motor change scores that could contribute to the variance in the play scores. These results certainly suggest there is a complex relationship among age, severity of impairment, sitting, and the development of play, which is worthy of further study.

One important contribution that this study makes to the literature is the extension of the PIECES system to assess play behavior in children with motor-delays. The present study is the first and only study ever conducted using the newly developed PACMI instrument. Although the scale itself, assessment procedure, and coding of behavior is well-grounded in prior research (Kelly-Vance and Ryalls, 2014), additional research is needed to further document the validity and utility of this scale. The development of play has been linked to development in numerous other cognitive and social domains. Thus, interventions that improve a child’s ability to play may be important to improving function in other areas. This study provides initial support for a reliable tool that can be used to measure the emergence of play skills in children with significant motor-delays.

This study, like most, suffers from limitations that leave additional questions unanswered. Most notably, all children were exposed to the same sitting intervention, therefore comparison with a control group is not possible. Thus, we cannot state with absolute certainty that the changes in sitting and play were not the result of maturational changes alone. However, we find this highly improbable, given the severity of the delays experienced by these children. Ultimately, however, it would be desirable to replicate the present study with a control group of children given no intervention. A second limitation concerns the fact that only a single type of sitting intervention was used. Therefore, while we can tentatively conclude that the sitting intervention was effective, we do not know if different types of sitting interventions would be more or less effective or have more or less of an effect on play. Additional research is needed comparing the effectiveness of different types of sitting interventions on both sitting and play. A third limitation concerns the lack of information about the children’s cognitive level. Apart from basic demographic information, the only thing known with any certainty was each child’s level of sitting ability. No data concerning level of cognitive functioning was collected or available. Anecdotally, a wide range in play ability was observed across the children in this study. Given that cognitive ability is linked to the complexity of play in typically developing children (Piaget, 1951), it would be interesting in future studies to systematically examine the relationship among motor ability, cognitive ability and play. However, measuring cognitive ability in these children is difficult, given their limitations. One potential window into cognitive ability may involve looking measures or eye tracking (Karatekin, 2007; Harbourne et al., 2013). Finally, a fourth possible avenue for future study concerns the effect of a play intervention on motor development. While this study suggests that improvements in sitting may lead to improvements in play, particularly for severely impaired children, an interesting question not addressed by the present study is if an intervention targeting play behavior in children with CP would have positive benefits on sitting. Playing may improve sitting because reaching for toys requires children to employ variable strategies to control posture and enable interaction with the toys (Harbourne and Kamm, 2015). If children are spontaneously motivated to engage in play, then interventions designed to improve play may also naturally have positive influence on a child’s ability to sit.

In summary, in spite of these limitations, the present study documents that emerging play-behavior can be reliably measured in motor-delayed children, that an ecological intervention can significantly improve sitting ability in children with moderate to severe CP, and that these improvements in sitting may lead to improvements in simple pretend play, particularly for more severely delayed children. This link between motor-development and play is consistent with views with ecological and systems theories that emphasize the significant influence that motor development and self-directed action can have on many areas of development including perception, cognition, emotional development, and others (Campos et al., 2000; Smith, 2005; Maruyama et al., 2014). Importantly, documenting a link between sitting and play in motor-delayed children demonstrates that such links can exist independent of typical chronological development.

## Author Contributions

All authors contributed to the following: substantial contributions to the conception or design of the work; or the acquisition, analysis, or interpretation of data for the work; drafting the work or revising it critically for important intellectual content; final approval of the version to be published; agreement to be accountable for all aspects of the work in ensuring that questions related to the accuracy or integrity of any part of the work are appropriately investigated and resolved.

## Conflict of Interest Statement

The authors declare that the research was conducted in the absence of any commercial or financial relationships that could be construed as a potential conflict of interest.

## Footnotes

1 http://www.plaisuno.com.

2 http://www.cdc.gov/ncbddd/cp/data.html
